# Behavior and Welfare of Undocked Heavy Pigs Raised in Buildings with Different Ventilation Systems

**DOI:** 10.3390/ani11082338

**Published:** 2021-08-08

**Authors:** Marika Vitali, Enrica Santolini, Marco Bovo, Patrizia Tassinari, Daniele Torreggiani, Paolo Trevisi

**Affiliations:** Department of Agricultural and Agri-Food Sciences and Technologies, University of Bologna, 40127 Bologna, Italy; marika.vitali4@unibo.it (M.V.); enrica.santolini2@unibo.it (E.S.); marco.bovo@unibo.it (M.B.); patrizia.tassinari@unibo.it (P.T.); daniele.torreggiani@unibo.it (D.T.)

**Keywords:** animal behavior, animal welfare, computational fluid dynamics, housing conditions, undocked tail, heavy pigs, animal-based measure, ventilation systems, association study, qualitative behavioral assessment

## Abstract

**Simple Summary:**

The relationship between animal welfare and housing conditions is still a matter of debate. The present study aimed to evaluate animal welfare of undocked heavy pigs from the same farm, raised in buildings with different ventilation systems, i.e., mechanical and natural, throughout the fattening period (90–160 kg average weight). Ventilation efficiency was evaluated using computational fluid dynamics. Results showed that overall pigs raised in the mechanical ventilated building were in a more positive affective state. Despite that, with hot temperatures, the higher occurrence of pig soiling indicated heat stress and consequent welfare impairment. The higher frequencies of dog sitting behavior also indicated worsening of welfare conditions in the middle–late phases of fattening, likely imputable to the lack of stimuli and boredom in the pigs.

**Abstract:**

The present study aimed to evaluate animal welfare of pigs from the same farm, raised with two ventilation systems. The study involved 60 pens of fattening pigs, raised in two buildings: one naturally ventilated (NV) and the other mechanically ventilated (MV). Pigs were assessed on three observation days: at 40 kg (T1), 100 kg (T2), and 160 kg (T3) of live weight. Animal-based measures were used such as qualitative behavioral analysis (QBA), behavioral measures (BMs), and lesion and health measures (LHMs). Housing conditions (HCs) measured at each observation day were the number of pigs per pen, space allowance, temperature, light, and CO_2_. The association study was performed using a general linear model and analysis of variance. Ventilation effect was analyzed by performing computational fluid dynamics. Results showed that overall pigs raised in the MV were in a more positive affective state. Despite that, with hot temperatures, the higher occurrence of pig soiling indicated heat stress in pigs and consequent welfare impairment. The higher frequency of pigs showing dog sitting behavior at T2 and T3 suggest welfare worsening in the last phases of fattening. The study concludes that ventilation system influences animal behavior and overall animal welfare, especially during the warmer season.

## 1. Introduction

Animal-based measures (ABMs) are considered the most reliable indicators to assess the welfare status of an animal and to identify the risk factors in the management and environmental conditions [1]. They include a large variety of indicators such as behavior, clinical signs (e.g., skin lesions, pathologies), and physiological and productive parameters. ABM_S_ allow measuring how a single animal (or a group of animals) reacts to environmental and management stressors. One of the main stressors in intensive pig herds is the fluctuation of the (indoor) environmental temperature, particularly when it overcomes the thermoneutrality threshold of the animal [2]. Further causes of environmental stress can be attributed to excessive relative humidity (rH) and harmful gas concentrations (e.g., CO_2_ and NH_3_) [3,4]. Concerning temperature control and gas removal, ventilation efficiency plays the main role, and it is mainly dependent on the ventilation system in the barn [5]. At present, pig barns in the Mediterranean area are mainly wind-driven and, thus, naturally ventilated, while mechanical ventilation systems are less common [6]. In naturally ventilated buildings, the external wind influences the indoor velocity magnitude and distribution [7]. In these systems, the accurate control of the indoor conditions is not always feasible, especially on warm days, when the windows and vents of the barns are fully open [8]. In mechanically ventilated barns, the ventilation efficiency can vary depending on the operational conditions of the ventilation system [9]. In both natural and mechanical ventilation systems, the ventilation efficiency can vary depending on the geometry of the piggery structure; therefore, it should be carefully evaluated.

Inadequate ventilation, leading to subsequent changes in temperature, humidity, and presence of gas and dust [10,11,12], has been found linked to pig behavior, health, and physiology [9]. For some ABMs, the effects of poor ventilation on animal health are well known; for example, an insufficient air exchange can increase the occurrence of respiratory disease and thermal stress [7,13], while contrasting results have been observed with regard to animal behavior. In the case of aggressive behaviors and outcomes such as lesions, specific studies are lacking. Some studies reported that heat stress can lead to the development of aggressive behavior and consequent skin lesions [14]; other works showed that high CO_2_ concentration might induce higher inactivity rates of pigs and could increase the risk of overloading with subsequent occurrence of lesions in the middle area of the body and/or the prevalence of bursitis due to the prolonged contact of bone prominence with the floor [10].

Furthermore, tail biting and relative tail lesions have been hypothesized to be influenced by the ventilation conditions (i.e., magnitude air velocity, air direction, and air exchange) and typologies (i.e., natural or mechanical). Hunter et al. [15] observed a higher presence of lesions in the case of mechanical ventilation than in the case of natural ventilation. Therefore, it is important to consider that, according to Hunter et al. [15], the natural ventilated building considered in the study was provided with straw litter, which is considered the “gold standard” to prevent tail biting behavior, and this aspect might have biased the results. On the contrary, the study by Scollo et al. [16] reported higher frequencies of tail lesions in the farms adopting natural ventilation.

Lastly, no studies have been found on the effect of ventilation on the emotional state of fattening pigs. Today, it is worth noting that animals need a positive emotional state to be in a positive welfare condition [17]; therefore, ABMs to assess pig emotional state have been proposed, such as qualitative behavioral assessment (QBA), tail posture, and tear staining [18,19]. It has been questioned if the impact of ventilation, dust, and air quality could be a confounding factor for some emotional state indicators [20,21]. It has been hypothesized that temperatures out of the acceptable range can influence tail posture, which is also an indicator of tail biting behavior in a group of pigs, and pig emotions, leading to misinterpretation of the measure itself. Similarly, high dust and gas concentrations can mislead the interpretation of tear staining as an indicator of emotion in pigs, due to inflammation of the eye and conjunctiva by poor air quality [20].

Lastly, previous research found evidence that the age and weight of the pigs might also influence their behavioral response toward housing conditions, temperature, and air quality [22], stressing the need to consider this variable when assessing the effect of ventilation on ABMs in a productive cycle.

Being able to identify the relationships between ABMs and ventilation conditions might be helpful to prevent the occurrence of harmful social behaviors and, therefore, increase pig welfare conditions.

This study hypothesized that there would be significant associations between housing condition management and behavior, lesions, and emotional state in undocked pigs during the fattening phase. Therefore, the first aim of the study was to quantify and qualify the main welfare issues of the pigs. The second aim was to define and compare the ventilation conditions in two different buildings of a case study farm, using computational fluid dynamics (CFD) simulations, and to investigate possible relationships between welfare indicators and housing and ventilation conditions, to evaluate how these can impact pig welfare.

## 2. Materials and Methods

In the study, a list of animal-based indicators of both negative and positive welfare status was tested in two groups of finishing pigs reared in a barn located in northern Italy. The two groups were housed in two different buildings, which were selected for the study because they have analogous management conditions but are characterized by different ventilation conditions (natural vs. mechanical ventilation).

### 2.1. Livestock, Building, and Animals

This study was conducted in a commercial pig farm located in northern Italy, in the heart of the so-called Food Valley. At the beginning of the observations, a face-to-face interview with the farmer was conducted by two authors, P.T. (Paolo Trevisi) and M.V., using a questionnaire (Supplementary File S1). The purpose of the questionnaire was used to determine the overall rearing condition of the farm and main management practices. Briefly, the farm was a fattening farm belonging to a specific supply chain. Therefore, all the pigs came from the same group, genetic line, and overall housing conditions. All pigs were left undocked. Pigs at the beginning of the fattening period weighted 40 kg and were raised for 4 months until 160 kg of live weight. The farm had three employees caring for the animals, and there were written procedures about the prevention of tail biting and emergency culling.

### 2.2. Description of the Building

**Figure 1:**
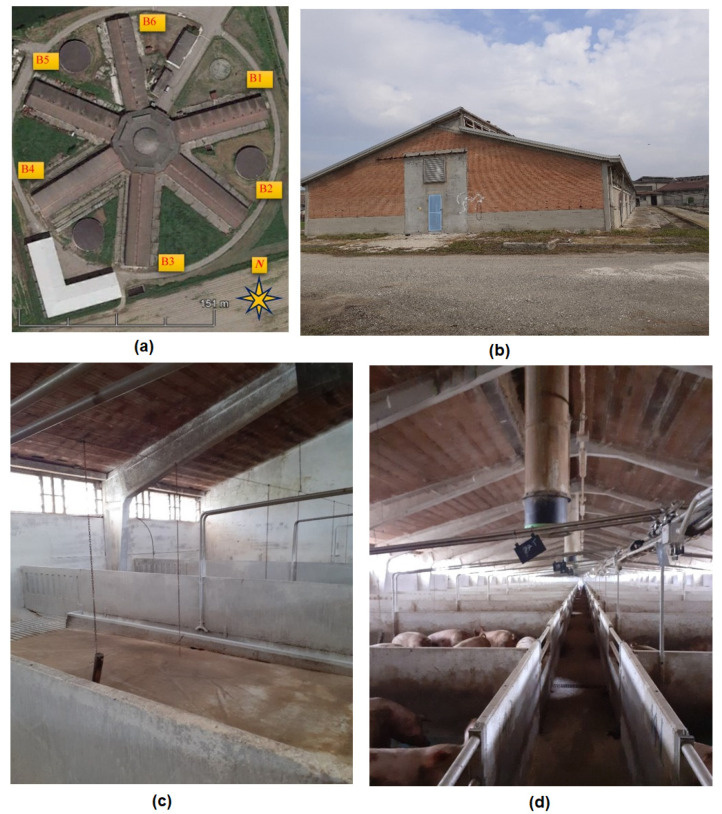
The case study building: (**a**) aerial view of the farm; (**b**) external view of building B3 (naturally ventilated); (**c**) internal view of building B3 (naturally ventilated); (**d**) internal view of the building B5 (mechanically ventilated).

The farm was originally designed to host dairy cows; however, in the 1990s, it was restored and converted to house finisher pigs. An aerial view of the particular shape of the building, similar to a “star” with six buildings labeled B1–B6, is shown in Figure 1 together with some representative pictures, both internal and external. The central core of the farm is a heptagon with an edge dimension of about 20 m; the inner height is 7.50 m at the eaves and 9.40 m at the top of the central dome. The buildings are 72.30 m long. Buildings B1, B2, B3, and B6 are 17.70 m wide, whereas B4 and B5 are 19.80 m wide.

The present study focused on B3 and B5. The two different buildings were selected since they can be considered representative of the two different ventilation conditions of the farm. B3 is naturally ventilated and is (north–south) oriented. Natural ventilation (NV) in B3 is obtained through wall windows and ridge vents running all along the building length. B5 is SE–NW oriented and is equipped with a mechanical ventilation system. The mechanical ventilation (MV) is realized by means of six longitudinally equally spaced chimney fans (Fancom, The Netherlands). Furthermore, the wall windows on the lateral longer side allow for the entering of the fresh air.

Each building hosts around 700 pigs (for a total of 4200 pigs in the farm). The buildings hosting the pigs are characterized by two lines of pens (19 + 19) with a central service corridor of about 0.90 m running along the whole length of the building. Pens have about 25% of the surface characterized by a slatted floor and 75% characterized by a full slab. Each pen is provided with a trough for liquid feed (provided twice a day) and two nipple drinkers. Environmental devices are constituted by a chain and a chain with wood placed in the middle of each pen.

### 2.3. Sampling Procedure and Investigated Scenarios

The present study involved a total of 60 pens, 30 in the NV building (B3) and 30 in the MV building (B5). The average number of pigs/pen during the study was 28.23 ± 2.77 SD. The animals in each building were assessed on three observation days (T), for a total of 1694 pigs observed:

- T1: 1 week after their allocation in the building, at about 40 kg of body weight;
- T2: 1 month after the allocation, at about 90 kg of body weight;
- T3: the day before loading to the abattoir, at about 160 kg of body weight.

At each observation point (i.e., observation day), 10 pens per building were randomly chosen, according to the Welfare Quality protocol [23].

The three observation days (T1, T2, and T3) for the two buildings (B3 and B5) led to six different scenarios in which data on housing conditions and data on animal-based measures were collected. Moreover, the same six scenarios were numerically investigated by means of CFD simulations.

### 2.4. Experimental Measures

#### 2.4.1. Animal-Based Measures

The ABMs were recorded on each observation day. Full references and explanations of the measures are reported in Table 1.

Measures were divided into qualitative behavior assessment (QBA), behavioral measures (BMs), and lesions and health measures (LHMs). The same observer (i.e., the co-author M.V., with 5 years of experience with ABMs and trained to use the Welfare Quality protocol) recorded all the data. QBA was observed between 9:00 and 10:00 am and consisted of four observations (5 min each) for a total of 20 min. Then, the data were reported on a 125 mm scale and multiplied by the coefficients indicated in the protocol [23] to calculate the QBA score. BMs were evaluated between 10:00 am and 11:00 am with the direct observation of all pigs in each pen, three times per pen, for 5 min per observation. Then, the average of the three observations was calculated. Behavioral measures consisted of two types of observation: categories of behavior as described in the Welfare Quality [23], and abnormal behaviors or stereotypies.

Categories of behavior included “inactive behavior”, “social behavior”, “exploratory behavior”, and “other active behavior”, as detailed in Table 1. The frequency of “social”, “exploratory”, and “other active behaviors” was determined by the total active behavior in each pen. The frequency of “inactive behavior” was calculated as a function of the total behavior observed, as explained in the Welfare Quality protocol for pigs [23]. Observed stereotypies and abnormal behaviors were recorded (i.e., tail biting, ear biting, dog sitting, bar biting), and they were calculated as the percentage of the mean of animals exhibiting the behavior (A_b_) over the total number of animals (A_tot_) in the pen [24].

A_b_/A_tot_ × 100 (%). (1)

Then, the sum of all stereotypies and abnormal behavior was calculated for each pen.

LHMs were assessed in the afternoon on a sample of 15 pigs/pen. The assessment was carried out inside the pen at a distance of 0.5 m from the pig, using a headlight when necessary. Only the left side of the pig was observed. Skin lesions were scored in each pig using a score [23] ranging from 0 to 2 (i.e., 0: up to 4 lesions, 1: from 5 to 10 lesions; 2: more than 11 lesions); then, the most frequent (i.e., prevalence) score was calculated and considered for each pen. The lesion score, ranging from 0 to 200 [24], was calculated for each monitored area as follows:

prevalence of lesion with a score of 1 + (2 × prevalence of lesion with score of 2).

The same formula was used to calculate the dirtiness and tear staining index.

Other LHMs were recorded using a Y/N score (where Y denotes presence, and N denotes absence), and the prevalence of pigs having a Y score was calculated in each pen [24].

#### 2.4.2. Housing Condition Measures

At each observation day, the most relevant parameters characterizing the housing conditions (HCs) were measured. These were light intensity, temperature, CO_2_ concentration, stocking density, and dustiness. Light intensity was measured using a Mini Light Meter (UNI-T UT383, Dongguan City, China). The temperature was recorded with a Datalogger (UNI-T UT330C USB, Dongguan City, China). CO_2_ concentration was measured with an IR sensor using a XAM8000 Multigas Detector (Dräger, Lübeck, Germany). The area of the pen was calculated using a Laser Distance Meter (Extech DT40M, Nashua, NH, USA), excluding the feeding area, and then divided by the number of pigs to obtain the stocking density. Light intensity, temperature, and CO_2_ were recorded at the pigs’ eye level as the average of three points in the pen: the corner closest to the center of the building, in the middle of the pen, and the opposite corner closer to the external wall.

#### 2.4.3. Statistical Analysis of HCs and ABMs

All statistical analyses were performed using R software [29]. The statistical unit was a pen. All observation days were considered separately. Descriptive analyses of ABMs were performed using the psyc.ir package [30]. Frequencies of behavior or LHM prevalence (considering the sum of scores of 1 and 2) showing a prevalence below 5% were not submitted to further statistical analyses. A general linear model (GLM) was carried out for the two buildings on the factors of the HCs, BMs, and LHMs intended as dependent variables using the building as a factor (independent variable). The GLM procedure was performed using the lme4 package [31], and the chi-squared test was used to evaluate the differences between the two buildings (lsmeans package, [32]). QBA descriptors were subjected to principal component analysis (PCA) using the FactoMineR package [33]. Statistical significance was set at *p* ≤ 0.05.

### 2.5. Computational Fluid Dynamics Simulations

The three-dimensional distribution of the ventilation conditions of buildings B3 and B5 was assessed using CFD simulations. The CFD simulations considered the model of the whole geometry of the pig farm, including the surrounding buildings, to take into account the interactions between the different structures. The geometrical model was developed in Autodesk Inventor [34], and the CFD analyses were carried out in VENTO AEC 2020 [35]. The geometrical model of the buildings is depicted in Supplementary Figure S1.

CFD analysis is based on the governing fluid dynamics equations (continuity, momentum, and energy). The general Navier–Stokes equation, with Boussinesq approximation that relates the Reynolds stresses and velocity gradients through the eddy viscosity, has the following form:

(2) $$\rho U_{j}\left(\frac{\partial U_{i}}{\partial x_{j}}\right)=\frac{-\partial P}{\partial x_{j}}+\frac{\partial }{\partial x_{j}}\left[\left(\mu +\mu_{t}\right)\frac{\partial U_{i}}{\partial x_{j}}\right].$$

These models are also called eddy viscosity models and are classified on the basis of the number of transport equations. The model, chosen for the simulations, was the two-equation *k–ε standard* model, where the equations for *k*, kinetic energy per unit mass of the turbulent fluctuations, and *ε*, dissipation rate, are as follows:

(3) $$\frac{\partial k}{\partial t}+U_{j}\frac{\partial k}{\partial x_{j}}=\frac{\mu_{t}}{\rho }S^{2}-\epsilon +\frac{\partial }{\partial x_{j}}\left[\frac{1}{\rho }\left(\mu +\frac{\;\mu_{t}}{\sigma_{k}}\right)\frac{\partial k}{\partial x_{j}}\right],$$

(4) $$\frac{\partial \epsilon }{\partial t}+U_{j}\frac{\partial \epsilon }{\partial x_{j}}=\frac{\epsilon }{k}\left(C_{1\epsilon }\frac{\mu_{t}}{\rho }S^{2}-C_{2\epsilon }\epsilon \right)+\frac{\partial }{\partial x_{j}}\left[\frac{1}{\rho }\left(\mu +\frac{\mu_{t}}{\sigma_{\epsilon }}\right)\frac{\partial \epsilon }{\partial x_{j}}\right],$$

where $\sigma_{k},\;\sigma_{\epsilon },C_{2\epsilon },\;\mathrm{and}\;C_{1\epsilon }$ are experimental constants available from the literature [36].

The boundary conditions (i.e., outdoor temperature, relative humidity, wind magnitude, and wind direction) for each one of the six scenarios considered were defined according to the data recorded by the weather station of ARPAE, placed in Rolo (RE), located only 5 km from the farm. They are summarized in Table 2. In the simulations, the reference wind velocity profile was defined by the following logarithmic profile:

(5) $$u\left(z\right)=\frac{u_{}}{K}log\left(\frac{z-d}{z_{0}}\right),$$

where u(z) is the average wind speed at height *z* above the ground, *u* is the friction velocity, K is the von Karman’s constant (assumed equal to 0.40), *d* is the displacement length, and *z*_0_ is the aerodynamic roughness (in m).

Moreover, for the building with mechanical ventilation, each chimney was defined in the model as a pressure–volume source with a pressure gradient of 16.8 Pa/m obtained from the datasheet of the fans (Fancom, The Netherlands). The mesh was selected after a grid independency study based on four different grids in terms of cell number. The final grid adopted for the analyses was characterized by 9 × 10^6^ million cells.

A preliminary experimental campaign was conducted on the farm to collect the air velocity magnitude using a hotwire anemometer (Delta Ohm, Italy) with an uncertainty of 0.01 m/s, to validate the numerical model for both buildings. The results of the validation process are shown in Supplementary Figure S2. The relative mean square error (RMSE) results were equal to 0.003 m/s for the natural ventilated case and 0.048 m/s for the mechanically ventilated building, confirming the limited difference between experimental and numerical results.

## 3. Results

During the study, clinical observations were carried out by the farm veterinary and the coauthors P.T. (Paolo Trevisi) and M.V.; no infective disease occurred, there was no need for antibiotic or other veterinary treatment, and the animals were in overall good health status.

### 3.1. Animal-Based Measures

#### 3.1.1. Qualitative Behavior Assessment

**Figure 2:**
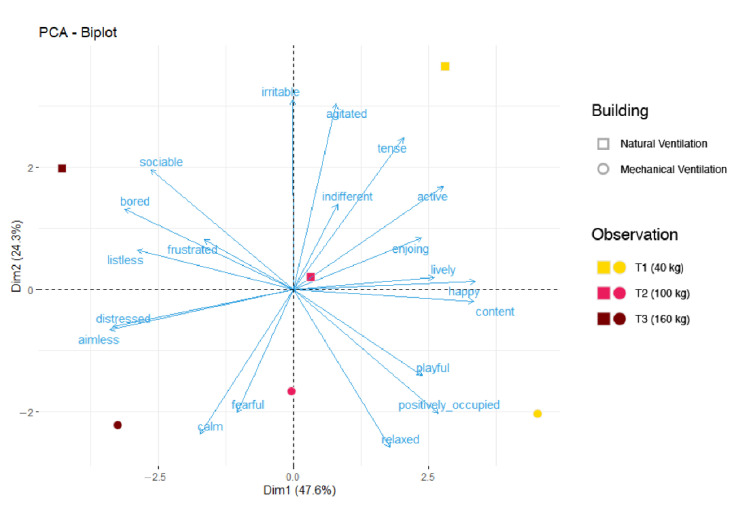
Qualitative behavior assessment (QBA) analysis performed on the pigs. The QBA was performed following the indications of the Welfare Quality [23]. Descriptors and factors were analyzed using principal component analysis. The results of Dim1 (Principal component dimension 1) and Dim2 (Principal component dimension 2) are reported. One spot corresponded to one observation. The color of the spot indicates the observation: T1 (yellow, indicating pigs of 40 kg on average); T2 (pink, pigs of 100 kg of average); T3 (red, pigs of 160 kg on average). The shape of the spot corresponds to the building: square = pigs raised in naturally ventilated building; circle = pigs raised in the mechanically ventilated building.

Considering the three observation days, the QBA score (average ± SD) resulted equal to 16.8 ± 2.2 and 27.8 ± 20.6, respectively, for building B3 with NV and building B5 with MV. The main difference was obtained in the first assessment (T1), where NV had a score of 17.8 and MV had a score of 51.07. The PCA analysis showed that the first and second dimensions (Dim) together explained 71.9% of data variance. Dim1 accounted for 47.6% and Dim2 accounted for 24.3% (see Figure 2).

Figure 2 shows that Dim1 accounted for the observation day, while Dim2 accounted for the building. The output of the PCA is reported in Table 3. In general, considering the two buildings (Dim2), NV showed overall higher arousal and negative emotional states (i.e., tense, irritable, agitated) than MV, where the animals showed more positive state and lower arousal signs. On the other hand, considering the effects of the observation time (Dim1), pigs were perceived as being in a more positive emotional state (i.e., active, relaxed, enjoying, playful, positively occupied, lively, content, happy) at T1 and a negative emotional state (i.e., bored, aimless, distressed and listless) at T3.

#### 3.1.2. Behavioral Measures

**Figure 3:**
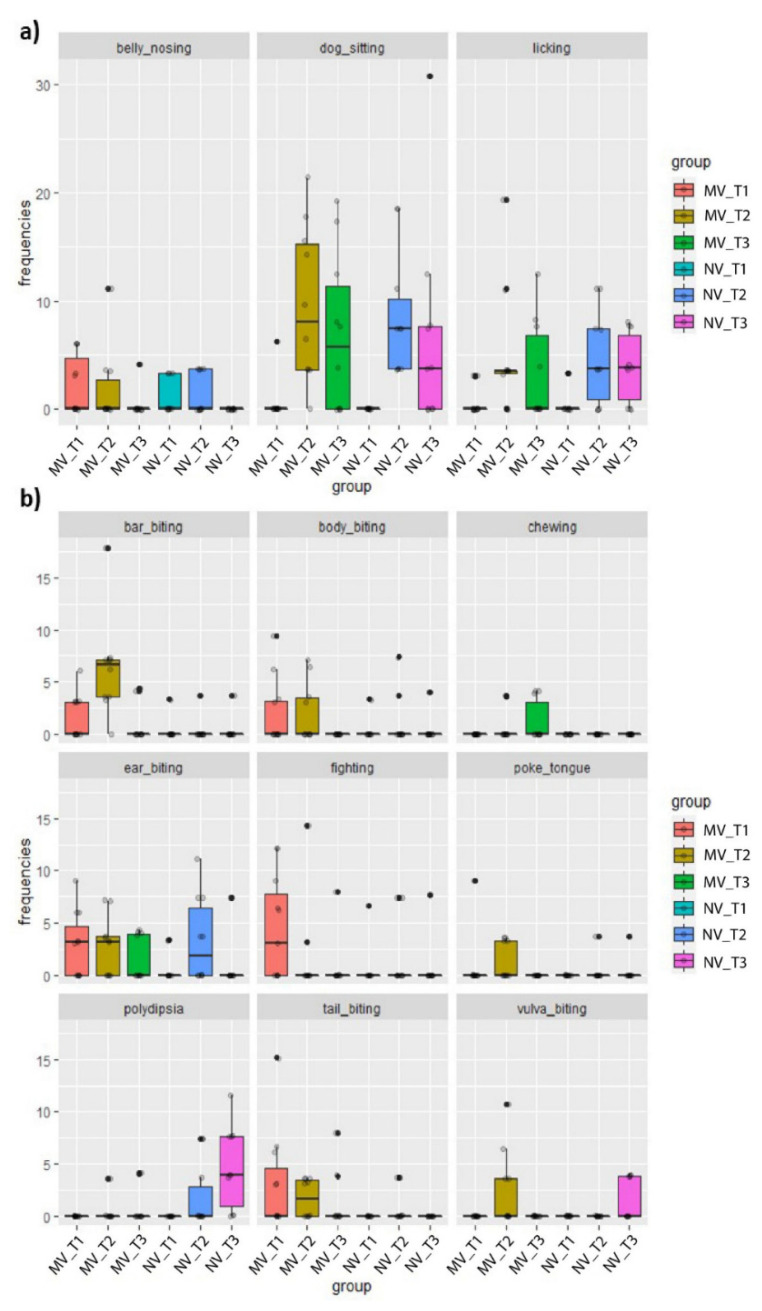
Observed stereotypies in the two buildings at different times. Each behavior was grouped in (**a**,**b**) according to the observed frequency.

**Figure 4:**
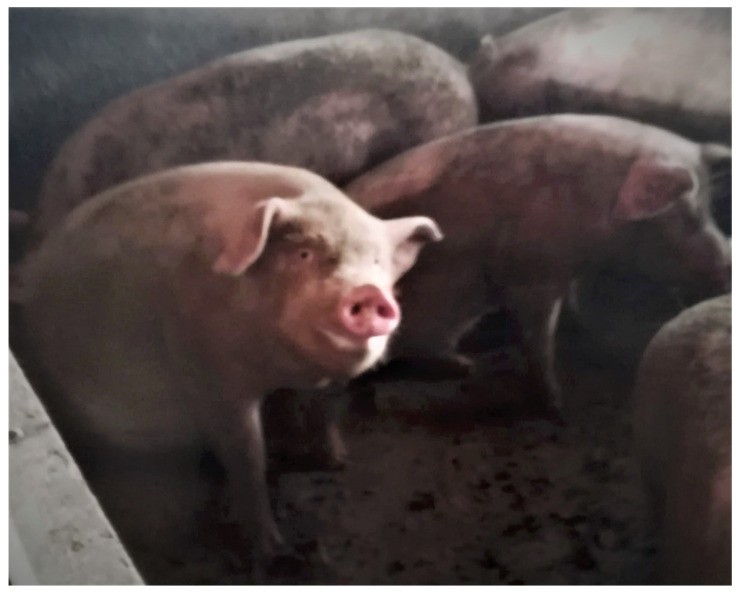
Example of dog sitting stereotypic behavior frequently observed during the behavioral assessment.

Different pig stereotypies were observed during the trial. They are summarized in Figure 3. The observed stereotypies show that belly nosing and tail biting were more frequent at T1 and T2, while dog sitting and licking were more frequently observed at T2 and T3, in both buildings. Dog sitting was the most evidenced stereotypy overall (see Figure 4).

Because the prevalence of single stereotypies was substantially low in many cases, they were summed and considered together for a statistical comparison between the two buildings. Statistical results from the behavioral analysis are reported in Table 4.

At time T1, piglets in the MV building had a higher prevalence of tail position, as compared with the NV case (*p* < 0.0001). Pigs in MV also showed higher stereotypies as compared to NV (*p* < 0.0001) and negative behavior toward pen mates (*p* = 0.02). Inactive behaviors were mostly observed in the NV building (*p* = 0.0002). At time T2, stereotypies still had higher frequencies in the MV building compared to NV (*p* = 0.046), while the other behaviors did not show substantial differences. At time T3, pigs in MV showed a higher prevalence of a hanging down tail position (*p* = 0.01), negative social behaviors (*p* = 0.04), and other active behavior (*p* = 0.03) compared to NV. Moreover, pigs in the MV were also more inactive than those from the NV (*p* = 0.004).

#### 3.1.3. Lesions and Health Measures

Only LHMs showing LSI with a score higher than 10 or with prevalence above 5% were considered in the analysis (see Table 5). The results showed that, at T1, pigs in the MV building had higher scores for tear staining and dirtiness compared to pigs in the NV building (*p* < 0.0001). Pigs in NV instead showed more tail lesions than those in MV (*p* = 0.01) and had a trend of higher front lesions (*p* = 0.07). At T2 and T3, no significant differences were observed in terms of LHMs. Only a slight trend (*p* = 0.09) was observed in front lesions, where NV pigs had a higher score than MV.

### 3.2. Housing Condition Measurements

The results concerning the housing condition measurements in the two buildings, at the time of observation, are reported in Table 6. The records of CO_2_ at T1 and temperature at T2 are missing due to technical issues during the assessment. The results show that light and temperature did not differ between buildings, except for T3. In fact, at T3, the temperature was rather high in both buildings but significantly higher in NV building pens compared to the MV case (*p* = 0.04), whereas the light was significantly lower in the MV case as compared to NV (*p* = 0.04), even if the value was higher than the minimum (i.e., 40 lux) reported in the Dir 120/2008 EC. The CO_2_ concentration was always below 3000 ppm (the level indicated by EFSA as dangerous for pigs [10]), but showed statistical differences at T2 and T3, with opposite trends. Specifically, at T2, the CO_2_ concentration was higher in the MV case compared to NV (*p* < 0.0001), while, at T3, the NV pens showed the highest CO_2_ concentrations (*p* < 0.0001). This outcome seems in line with the number of pigs per pen, which, in the first two observations, was higher in MV vs. NV (*p* < 0.0001 at T1 and *p* = 0.0003 at T2), while, at T3, it was higher in NV compared to MV (*p* = 0.02). Space allowance was higher in MV as compared to NV (*p* < 0.0001 at T1 and T2), except for T3 where no significant differences were observed between buildings. The space allowance accomplished the minimum standards required by the legislation in each observation day.

### 3.3. Computational Fluid Dynamics Simulations

Six simulations were performed in order to evaluate the indoor ventilation conditions (numerical scenario) on the six observation days in which housing conditions and animal-based parameters were measured: three simulations were set to analyze the air velocity magnitude in the NV building and three were solved for the study of the ventilation scenarios in the MV building. The simulations considered the different external conditions (i.e., wind velocity and wind direction, air temperature, and air relative humidity rH) of the relevant observation day (see Table 2).

**Figure 5:**
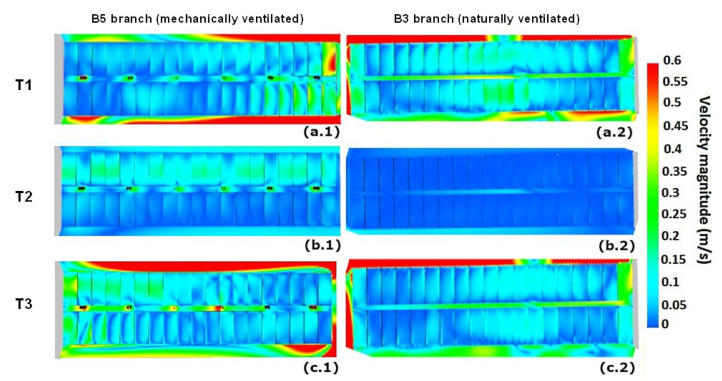
Indoor air velocity distribution 0.5 m above the pavement for different observation days (T), for mechanically ventilated building B5 ((**a.1**) at T1; (**b.1**) at T2; (**c.1**) at T3) and naturally ventilated building B3 ((**a.2**) at T1; (**b.2**) at T2; (**c.2**) at T3).

A qualitative comparison of the results is shown in Figure 5. It is possible to observe that indoor airflow distribution was substantially different between the two buildings, in terms of both airflow pattern and air velocity magnitude.

At T1, the two buildings showed very different indoor ventilation conditions. The mechanically ventilated B5 building (see Figure 5(a.1)) presented an air velocity magnitude highly variable with the length, with very low air velocity close to the central body and progressively increasing toward the opposite extremity, with a velocity peak of 0.6 m/s. On the contrary, in the NV building (see Figure 5(a.2)), results show that, in the central portion, the indoor air velocity ranged between 0.1 m/s and 0.2 m/s, while air velocity decreased in the two lateral portions, close to the extremities, of the building.

At T2 the outdoor configurations had similar air velocity magnitude and similar blowing wind direction in the two buildings. It is clear that the presence of the mechanical ventilation system in B5 (see Figure 5(b.1)), as expected, increased the indoor air velocity magnitude, while, in the natural ventilation case, the wind velocity was in general very low (see Figure 5(b.2)).

Similar conditions also characterized T3 of building B3, naturally ventilated (see Figure 5(c.2)). Instead, in building B5, the ventilation system resulted in the airflow distribution and magnitude being very inhomogeneous along the building length (see Figure 5(c.1)) compared to T1 and T2. This confirms the remarkable inhomogeneity of the internal ventilation condition between the different areas of the B5 building.

Further details of the air velocity magnitude are shown in Table 7.

As the table shows, during the monitored period, the indoor velocity magnitude in the MV building was, overall, more homogeneous than the air velocity in the NV building. Moreover, the average value was 0.10–0.11 m/s for MV, while it was just 0.06–0.07 m/s for NV.

## 4. Discussion

This study quantified and qualified the main welfare issues of pigs raised in two different buildings of the same farm. The ventilation strategy, as assessed by the CFD simulations, showed remarkable variability in the ventilation conditions of each building across the three observation days.

Overall, the QBA assessment showed that animals in the mechanically ventilated pens were in a more positive affective state, in accordance with the higher ventilation performance of the MV building, characterized by higher indoor air velocity. The QBA also evidenced a worsening in the affective states increasing with the age of the pigs. This last effect might also depend on the reduction in space allowance and the increase in temperature during summer, as well as changes in pig physiology, as previously reported [37,38,39]. Therefore, the comparison between the two buildings was performed separately for each observation time (i.e., T1, T2, and T3).

At T1, pigs in the MV group showed lower tail LSI compared to NV (Table 5) and a higher proportion of pigs with tail position up (Table 4). Tail lesions are the outcome of tail biting behavior. Tail biting is currently considered an iceberg indicator of poor welfare, having a negative effect on the emotive state of pigs [40]. Tail biting is an abnormal behavior, and its occurrence has been found to be strongly dependent by many managerial and environmental factors [41]. In accordance with the result of this study, Lahrmann et al. [27] proposed that assessing tail position would allow quickly identifying tail-bitten pigs since these pigs would keep a low tucked tail, while pigs which show few or no tail lesions would keep the tail curled and “up”.

In contrast, the behavioral analysis showed a higher frequency of negative social behavior in the group in the MV building, as well as higher stereotypy frequency. Despite that, lesion outcomes were not significantly different between the two animal groups in the different buildings. A discrepancy between these two indicators (behavior and lesion) was previously observed in other studies [24]. A possible explanation is that the lesions are the consequence of negative social behavior that occurred in a range of time (days or weeks), while the behavioral analysis in this study was a picture of the exact moment of the assessment since they were recorded by direct observation. Moreover, a limit of the present study was that behavioral analysis was carried out using direct observation; thus, although the behaviors were recorded in the same range of time, the observations were not conducted simultaneously. Pigs in the MV building showed an indeed higher score in tear staining and dirtiness, as compared to the NV building. Tear staining is the presence of a red stain in the left eye of a pig, as a consequence of the production of a red pigment by the eye pituitary gland. In pigs, it has been proposed as an indicator of negative emotional state because of a correlation with processing negative emotions [20,42]. Other studies have hypothesized that tear staining might also be stimulated by excessive gas concentration, dustiness, pen soiling, or other environmental conditions [24,43]. On this observation day, the MV building group showed a higher proportion of dirty pigs. Pig soiling has been frequently linked to higher gases in manure [44], and it might explain tear staining. The indoor air velocity was similar in the two buildings (Table 7); however, the higher number of pigs/pen with lower space allowance in the MV building compared to NV at T1 might also have enhanced this mechanism.

At time T2, behavior and lesions did not show any differences, except for overall higher stereotypies in the pigs in the MV building, mainly due to the percentage of pigs showing ear biting behavior. Similar to tail biting, ear biting has been considered an indicator of poor welfare so far [45]. Among the predisposing factors for ear biting, air quality has been reported to influence its occurrence [46]. In MV building, the results showed a higher concentration of CO_2_ as compared to NV building. CO_2_ is a product of respiration, which is heavier than oxygen; therefore, it has been found to fluctuate at the pig level. It is likely to presume that, on this observation day, the inhomogeneity of the airflow and speed was not efficient to remove CO_2_. Moreover, CO_2_ was found to be highly related to the number of animals. The MV building had one more pig per pen and showed a lower space allowance, contributing to an increase in the CO_2_ indoor concentration. This result might explain the higher presence of ear biting in this group.

Behavioral analysis evidenced also a high proportion of pigs showing dog sitting behavior on T2 and T3 observation days, in both buildings. Dog sitting has been considered a non-aggressive stereotypic behavior and an indicator of suboptimal welfare in pigs [47,48]. According to the study by Scollo et al. [49], pigs reared in intensive conditions increased the frequency of sitting behavior when space allowance decreased, e.g., in the fattening phase. This has been interpreted as the lack of space to lie down [49] or the consequence of boredom, leading to severe cognitive deprivation due to the barren environment [50,51]. A combination of the two factors might explain the results of the present study. Heavy pigs have a very restricted area available at the end of the cycle (because the current legislation states that pigs above 110 kg require min 1.00 m^2^, and, in heavy pig production, pigs can reach up to 180 kg at the end of the rearing period). Moreover, the behavioral analysis showed that the enrichment devices available to the pigs (metal chain and a metal chain with wood) were of marginal interest since pigs spent most of their time exploring the pen and very little time on the enrichment devices. Exploring the pen (over-exploring) has been considered another sign of boredom and poor welfare in intensive pig farms since the pens are usually in barren environments that do not provide cognitive stimuli to the pigs [52].

When considering T3, behavioral analysis evidenced a higher frequency of low tail position and negative social behavior in MV compared to NV. A low tail position has been previously associated with tail lesions; however, at this assessment, no differences were observed for tail LSI. It is important to consider that, at T3, the two buildings raised the maximum score in dirtiness, corresponding to almost all pigs in each pen having manure on >50% of the body surface; therefore, this condition might have biased the results from the lesion assessment. Pig soiling is considered the outcome of abnormal eliminative behavior in pigs [44]. Normally, pigs on a partially slatted floor tend to release urine and/or feces on the slatted floor and rest on the full floor. When certain predisposing factors occur (see later), pigs can develop abnormal behavior, which leads to pen and pig soiling. One of the main identified factors is thermal discomfort [53]; in fact, with high temperature, pigs raised indoor tend to rest on the slatted floor and release urine and/or feces on the full floor [54]. In very severe heat stress conditions, pigs tend to release urine and/or feces, as well as rest, on the full floor with the purpose of heat loss [37]. This latter condition has been considered an indicator of poor welfare since, in normal conditions, pigs prefer to avoid contact with their excreta [44]. The optimal temperature range for heavy pigs (140–180 kg of live weight) is estimated to be 18–20 °C. Therefore, at T3, the temperatures in the two buildings were very challenging for the pigs (29–30 °C on average), and neither type of ventilation was able to significantly reduce this temperature. The indoor ventilation was consistently different at T3. The MV building showed high air velocity in the extremities, compared to the central zone. On the other hand, the NV building showed homogeneous low air velocity throughout the building length. This difference could have affected CO_2_ concentration measured, which was significantly higher in the NV building as compared to MV. Accordingly, in the NV pens, higher frequencies of polydipsia were observed. Polydipsia is a stereotypy that can occur when pigs are submitted to heat stress, in an attempt to cope with hot temperatures [55]. Moreover, behavioral analysis observed also significantly lower inactive behavior in NV pens compared to MV ones. Housing conditions also revealed that temperature, light, CO_2_, and number of pigs per pen were higher in the NV case compared to MV. Those factors might have influenced the pigs’ behavior. Some studies have observed an increase in activity and aggression in the presence of high temperatures, due to heat stress and difficulty in finding a comfortable place to rest for pigs kept under intensive conditions [14,44]. In the present study, negative social behavior did not differ at T3, while a trend of more front LSI in NV pens was observed. Other studies, in contrast, observed an increase in lying behavior at high temperatures [37]. The difficulty in finding a lying place could be exacerbated when the number of pigs per pen increases, as in the NV pen group. Similarly, some studies reported that increasing illumination in the pig farms can lead to an increase in activity, which does not impair pig welfare [56]. In accord with the results, CO_2_ concentration was found to be directly proportional to pig activity by Zong et al. [57].

When the temperatures in the two building buildings were challenging, the higher air velocity in MV pens, even if not able to decrease the indoor temperature, could have contributed to a reduction in the heat perception at the pig level, as well as to a reduction in CO_2_ concentration, thereby influencing pig behavior and contributing to improving their welfare [10]. One limitation of the study was that the measurements could not be performed on the same day in both buildings, due to the farm flow chart, according to commercial agreements between farmers and buyers. However, this is the first study aimed at integrating ABM assessments and environmental measures provided by CFD simulations in heavy pigs. These preliminary results pose new questions regarding the effect of the interplay between outdoor and indoor conditions and ventilation systems on pig welfare, which will be further investigated.

## 5. Conclusions

This study pointed out that the indoor environment might influence animal behavior and overall animal welfare, and a detailed dynamic analysis of the indoor ventilation and outdoor wind and exposition is very important to improve the conditions in which the animals live and to identify the main risk factors that might impact animal health and welfare. Especially in the presence of hot temperatures, the high occurrence of pig soiling indicates severe heat stress in pigs and consequent welfare impairment. The high number of pigs showing dog sitting behavior also suggested welfare deterioration for the pigs, especially in the later phases of fattening, probably due to the combination of an absence of stimuli and heat stress. According to the results reported in this study, in hot climates, mechanical ventilation systems may not be sufficient to mitigate heat stress in pigs, and other solutions (e.g., cooling systems or water sprinklers) should be proposed to avoid welfare consequences for pigs.

## Figures and Tables

**Table 1:** List of parameters measured in the study, as well as their level of sampling, references, and description.

Type	Parameter	Reference	Description
QBA	Qualitative Behavior Assessment	[23]	The value was expressed in mm on a scale of 125 mm (visual analogue scale for QBA).
BM	Social behavior (negative and positive)	[23]	Modified from the reference. Negative social behavior included any aggressive social behavior or biting causing a response from the disturbed animal. Positive social behavior consisted of sniffing, licking, play, and moving gently away from the other animal without an aggressive or fight reaction from this individual. Negative and positive social behaviors were recorded, and they were expressed as the ratio of the percentage of social behavior (positive or negative) to the percentage of total active behavior (sum of social, explorative, and other behaviors).
BM	Explorative behavior (pen and environmental enrichment—directed)	[23]	Pen- and enrichment- directed exploratory behaviors were recorded, and they were expressed as the ratio of the percentage of social behavior (positive or negative) to the percentage of total active behavior (sum of social explorative and other behaviors).
BM	Other active behavior	[23]	Any active behavior not included in the previous categories.
BM	Inactive behavior	[23]	Any behavior when the animal remained motionless, i.e., without any activity.
BM	Tail biting	[24]	A pig attempting to manipulate or bite the tail of a pen mate.
BM	Ear biting	[24]	A pig attempting to manipulate or bite the ear of a pen mate.
BM	Body biting	[24]	A pig attempting to manipulate or bite a part of the body of a pen mate (e.g., flank, legs).
BM	Fighting	[24]	A pig involved in fighting.
BM	Bar biting	[25]	A pig biting or nibbling the bars or other structures in the pen.
BM	Belly nosing	[26]	A pig is performing the same movements as when nursing on the body of another pig.
BM,	Vulva biting	-	A pig is attempting to manipulate or bite the vulva of a pen mate.
BM	Poke tongue	-	A pig sitting or standing and poking the tongue in and out.
BM	Chewing	[25]	A pig showing continuous chewing without evidence of food in the oral cavity.
BM	Dog sitting	-	A pig sitting immobile on forelegs with hindquarters on the floor.
BM	Licking	-	A pig whose snout or tongue was used to touch a pen mate followed by head movements.
BM	Polydipsia	-	Repeated access of a pig to a drinker, with water intake that appeared excessive for its physiological needs and/or with water waste.
BM	Tail position	[27]	Scores were defined as follows: 0 = tail up (curly); 1 = tail down (hanging); 2 = tail tucked low (down and tucked to the body).
LHM	Skin lesions	[23]	Considering 5 separate areas (ear, fronts, middle, hindquarters, legs). Scores were defined as follows: 0 = up to 4 lesions visible; 1 = 5–10 lesions visible; 2 = 11 to 15 lesions visible.
LHM	Tail lesions	[23]	Modified from the reference as follows: 0 = absence of lesions; 1 = superficial biting along the length of the tail but no evidence of swelling or blood; 2 = fresh blood visible on the tail, or presence of a scar, swelling, or missing part of the tail.
LHM	Tear staining	[20]	Presence of red tears in the left eye. Modified from the reference as follows: 0 = absence of staining; 1 = staining barely detectable or less than 50% of the total eye area; 2 = staining up to 100% of the eye area or extending below the mouth.
LHM	Hernia	[23]	Modified from the reference. The presence or absence of this parameter was assessed in each observed individual.
LHM	Lameness	[23]	Modified from the reference. The presence or absence of this parameter was assessed in each observed individual.
LHM	Further care	[28]	Animals that had to be removed from the pen, needing further care, or being emergency culled. The presence or absence of this parameter was assessed in each observed individual.

QBA = qualitative behavior assessment; BM = behavioral measure; LHM = lesion and health measure.

**Table 2:** Dates and main outdoor characteristics collected for every one of the six scenarios considered in the CFD simulations.

Time	dd/mm/yyyy	T (°C)	rH (%)	V (m/s)	Dir (°)
Building B3—Natural Ventilation					
T1	02/04/2019	19.3	43.3	2.97	75
T2	21/05/2019	22.3	58.1	0.93	275
T3	20/08/2019	32.8	42.3	2.5	67
Building B5—Mechanical Ventilation					
T1	14/02/2019	10.6	49.3	3.01	263
T2	22/03/2019	17.1	31.1	1.07	270
T3	17/06/2019	29.9	46.6	2.01	80

T = temperature; rH = relative Humidity; V = air velocity; Dir = direction of the wind.

**Table 3:** Eigenvalue (Coordinate), quality of the representation (Cos2) and contribute of the descriptors (Contribute) used in the Qualitative Behavior Assessment.

Descriptors	Dim1			Dim2		
	Coordinate	Cos2	Contribute	Coordinate	Cos2	Contribute
Active	0.78	0.61	6.45	0.48	0.23	4.69
Relaxed	0.50	0.25	2.68	−0.73	0.54	11.04
Fearful	−0.30	0.09	0.92	−0.57	0.33	6.74
Agitated	0.22	0.05	0.52	0.86	0.74	15.23
Calm	−0.49	0.24	2.55	−0.67	0.45	9.28
Indifferent	0.23	0.05	0.55	0.40	0.16	3.21
Frustrated	−0.47	0.22	2.30	0.23	0.05	1.09
Enjoying	0.67	0.45	4.72	0.24	0.06	1.15
Bored	−0.89	0.78	8.24	0.37	0.14	2.88
Playful	0.68	0.46	4.79	−0.40	0.16	3.28
Positively occupied	0.76	0.58	6.06	−0.57	0.33	6.77
Lively	0.74	0.54	5.68	0.06	0.00	0.06
Sociable	−0.75	0.56	5.90	0.56	0.31	6.35
Irritable	−0.01	0.00	0.00	0.88	0.77	15.92
Tense	0.57	0.33	3.47	0.70	0.50	10.18
Aimless	−0.96	0.93	9.75	−0.19	0.03	0.71
Distressed	−0.95	0.90	9.46	−0.17	0.03	0.61
Content	0.95	0.89	9.38	−0.06	0.00	0.06
Happy	0.95	0.91	9.51	0.04	0.00	0.03
Listless	−0.82	0.67	7.06	0.18	0.03	0.69

Dim1 = Principal component dimension 1; Dim2 = Principal component dimension 2.

**Table 4:** Behaviors observed in the study in the two buildings.

Behavior Measure	UM	NV		MV		Estimate	*p*-Value
		Mean	SD	Mean	SD		
T1 (40 kg)							
Tail position up	%	33.7	6.3	77.9	19.7	−1.0	<0.0001
Hanging down tail	%	7.8	5.3	14.5	9.8	−5.0	0.1858
Tucked low tail	%	8.5	6.3	3.0	4.6	0.8	0.1596
Stereotypies	%	5.7	4.2	18.4	6.7	−12.7	<0.0001
Negative social behavior	%	4.1	3.9	11.0	3.9	−1.1	0.0147
Positive social behavior	%	2.1	3.0	1.2	1.9	0.8	0.5784
Pen exploration	%	74.1	13.2	75.6	3.8	−1.0	0.6607
Enrichment exploration	%	16.0	9.8	10.4	2.5	1.0	0.6607
Other active behavior	%	3.7	7.9	1.7	2.1	0.6	0.2541
Inactive behavior	%	64.3	14.7	48.0	9.6	16.9	0.0002
T2 (100 kg)							
Tail position up	%	87.3	9.1	84.7	7.7	1.0	0.4801
Hanging down tail	%	8.0	6.1	10.7	5.6	−1.0	0.3360
Tucked low tail	%	4.7	5.5	4.0	5.6	1.0	0.7913
Stereotypies	%	24.4	11.6	34.0	9.7	−9.5	0.0458
Negative social behavior	%	6.5	4.7	6.6	3.4	0.9	0.9438
Positive social behavior	%	6.0	5.8	4.7	6.0	−1.0	0.6459
Pen exploration	%	70.3	13.4	74.0	9.2	−3.7	0.4680
Enrichment exploration	%	9.2	4.3	11.3	2.8	−2.1	0.1999
Other active behavior	%	8.0	11.7	3.3	3.9	0.8	0.1983
Inactive behavior	%	54.8	12.8	54.0	11.0	0.9	0.8719
T3 (160 kg)							
Tail position up	%	97.3	5.6	94.0	7.3	1.0	0.2565
Hanging down tail	%	2.0	4.5	3.3	3.5	1.0	0.0111
Tucked low tail	%	0.7	2.1	2.7	4.7	1.2	0.5370
Stereotypies	%	19.3	12.2	16.3	10.3	3.0	0.5434
Negative social behavior	%	1.5	2.1	6.8	6.0	0.5	0.0408
Positive social behavior	%	2.7	2.3	4.8	7.0	1.2	0.3060
Pen exploration	%	77.7	15.4	78.9	12.4	1.0	0.8505
Enrichment exploration	%	5.4	4.4	7.1	9.0	1.0	0.5658
Other active behavior	%	12.7	13.6	2.4	3.1	0.7	0.0337
Inactive behavior	%	52.4	19.0	76.4	14.0	1.0	0.0043

UM = unit of measurement; NV = naturally ventilated building; MV = mechanical ventilated building.

**Table 5:** Lesions and health measures observed in the study in the two buildings.

LMI	UM	NV		MV		Estimate	*p*-Value
		Mean	SD	Mean	SD		
T1 (40 kg)							
Ear LSI	0–200	29.6	22.9	33.9	20.3	1.0	0.5847
Front LSI	0–200	18.1	9.1	11.5	10.4	7.7	0.0749
Tail LSI	0–200	24.8	11.4	11.5	12.3	13.7	0.0096
Tear staining	0–200	1.7	3.2	60.7	21.2	−59.0	<0.0001
Dirtiness	0–200	16.0	23.6	110.0	73.8	−94.0	<0.0001
T2 (100 kg)							
Ear LSI	0–200	43.3	39.7	42.0	21.8	1.0	0.9252
Front LSI	0–200	24.7	25.2	32.0	10.8	1.0	0.4498
Middle LSI	0–200	11.3	14.4	14.7	8.2	1.0	0.5627
Hindquarter LSI	0–200	13.3	16.6	11.3	10.4	1.0	0.7417
Tail LSI	0–200	27.3	14.6	34.7	15.0	1.0	0.2790
Dirtiness	0–200	119.3	78.2	118.0	40.9	1.0	0.9617
T3 (160 kg)							
Ear LSI	0–200	14.7	17.2	18.7	13.6	1.0	0.5647
Front LSI	0–200	26.0	13.5	14.0	13.1	1.0	0.0900
Middle LSI	0–200	14.0	13.9	18.0	15.1	0.2	0.3740
Tail LSI	0–200	6.0	8.6	10.7	12.3	1.1	0.3524
Dirtiness	0–200	200.0	0.0	192.7	15.2	1.0	0.1349

UM = unit of measurement; NV = naturally ventilated building; MV = mechanical ventilated building.

**Table 6:** Main data on the housing conditions obtained from the measurements realized during the monitoring period.

Housing Conditions	UM	NV		MV		Estimate	*p*-Value
		Mean	SD	Mean	SD		
T1 (40 kg)							
Pig per pen	pigs	30	0	32	1	1	<0.0001
Space allowance	m^2^/pig	0.9	0.0	0.8	0.0	0.9	<0.0001
Temperature	°C	21.7	0.6	20.6	1.8	1.0	0.1744
Light	lux	138.7	60.5	101.9	81.6	32.7	0.3900
CO_2_	ppm	941.7	117.9	-	-	-	-
T2 (100 kg)							
Pig per pen	pigs	27	0	29	2	1	0.0003
Space allowance	m^2^/pig	1.0	0.0	0.9	0.1	0.9	<0.0001
Temperature	°C	-	-	20.7	0.8	-	-
Light	lux	121.9	58.0	133.0	76.5	−11.1	0.7154
CO_2_	ppm	898.8	287.7	1310.8	141.4	−402.0	<0.0001
T3 (160 kg)							
Pig per pen	pigs	26	1	25	1	1	0.0168
Space allowance	m^2^/pig	1.0	0.0	1.0	0.0	1.0	0.1334
Temperature	°C	30.2	0.4	29.8	0.6	0.4	0.0422
Light	lux	179.5	123.5	107.3	30.1	1.0	0.0375
CO_2_	ppm	834.2	61.7	550.0	97.9	284.0	<0.0001

UM = unit of measurement; NV = naturally ventilated; MV = Mechanically ventilated.

**Table 7:** Indoor mean air velocity magnitude (V) obtained from the CFD simulations at a level of 0.50 m from the pavement, for the pens on the left and on the right of the central corridor, for buildings W3 and W5.

Time	dd/mm/yyyy	V_mean,left_ (m/s)	V_mean,right_ (m/s)
B3—Natural Ventilation			
T1	02/04/2019	0.090	0.106
T2	21/05/2019	0.007	0.005
T3	20/08/2019	0.059	0.083
B5—Mechanical Ventilation			
T1	14/02/2019	0.091	0.103
T2	22/03/2019	0.124	0.122
T3	17/06/2019	0.123	0.110

## Data Availability

All the data are provided within the manuscript and supplementary files.

## Supplementary Materials

The following are available online at https://www.mdpi.com/article/10.3390/ani11082338/s1: File S1. Farm questionnaire; File S2. Dataset; Figure S1. The geometrical model used in the CFD simulations; Figure S2. Validation of the numerical model used for the CFD simulations.
