# Supplementary material for: Behavior and Welfare of Undocked Heavy Pigs Raised in Buildings with Different Ventilation Systems
Source: Animals (Basel). 2021 Aug 8;11(8):2338. doi: 10.3390/ani11082338 (PMC8388702; doi:10.3390/ani11082338)
Supplement: Supplementary file 1 [file animals-11-02338-s001.zip › S1 file_ farm questionnaire.pdf]

Date:

Farm:

Breeding stage: Fattening (40 to 160 kg on average)

Breed and/or genetics:

Presence of pigs with undocked tail:

N.	notes

Number of employees

- ☐ **0**= 1 employee / < 2000 animals;
- ☐ **1**= 1 employee / 2000-4000 animals;
- ☐ **2**= 1 employee / > 4000 animals

Education level of owner and employees

- ☐ **0**= at least five years of experience or educational qualifications and periodic training courses on animal welfare and practical instructions to all employees;
- ☐ **1**= at least five years of experience or educational qualifications and training courses on animal welfare in the last three years and practical instructions to all employees;
- ☐ **2**= less than five years of experience and no educational qualifications and/or no welfare training course or not even a single employee presence without practical instructions received.

Procedures in case of tail biting outbreak:

- ☐ **0=** presence of written procedures and the employers were trained;
- ☐ **1**= no written procedure buy the employee were trained;
- ☐ **2=** absence of both

Emergency culling procedure:

- ☐ **0=** presence of written procedures and the employees were trained;
- ☐ **1**= no written procedure buy the employees were trained;
- ☐ **2=** absence of both

Feeding:

Water source:

Ventilation:

- ☐ Natural
- ☐ Artificial
- ☐ Mixed

Notes:
