# Supplementary figures and images for: Behavior and Welfare of Undocked Heavy Pigs Raised in Buildings with Different Ventilation Systems
Source: Animals (Basel). 2021 Aug 8;11(8):2338. doi: 10.3390/ani11082338 (PMC8388702; doi:10.3390/ani11082338)

**Supplementary Figure S1.** The geometrical model used in the CFD simulations.

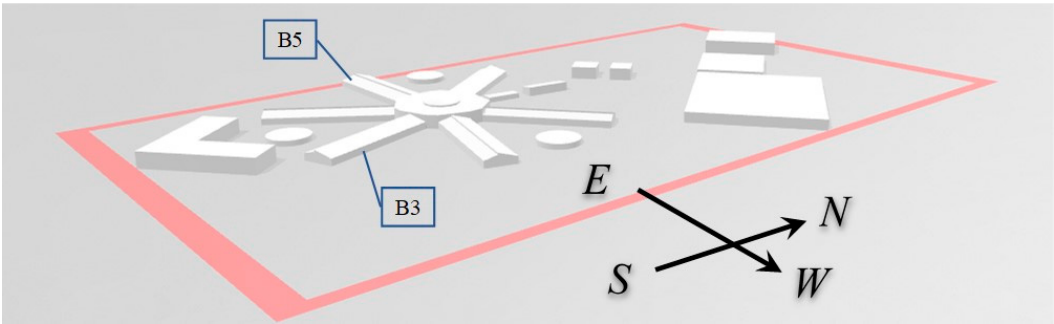

Supplement: Supplementary file 1 [file animals-11-02338-s001.zip › Supplementary figure S1.pdf]

Supplementary Figure S2. Validation of the numerical model used for the CFD simulations.

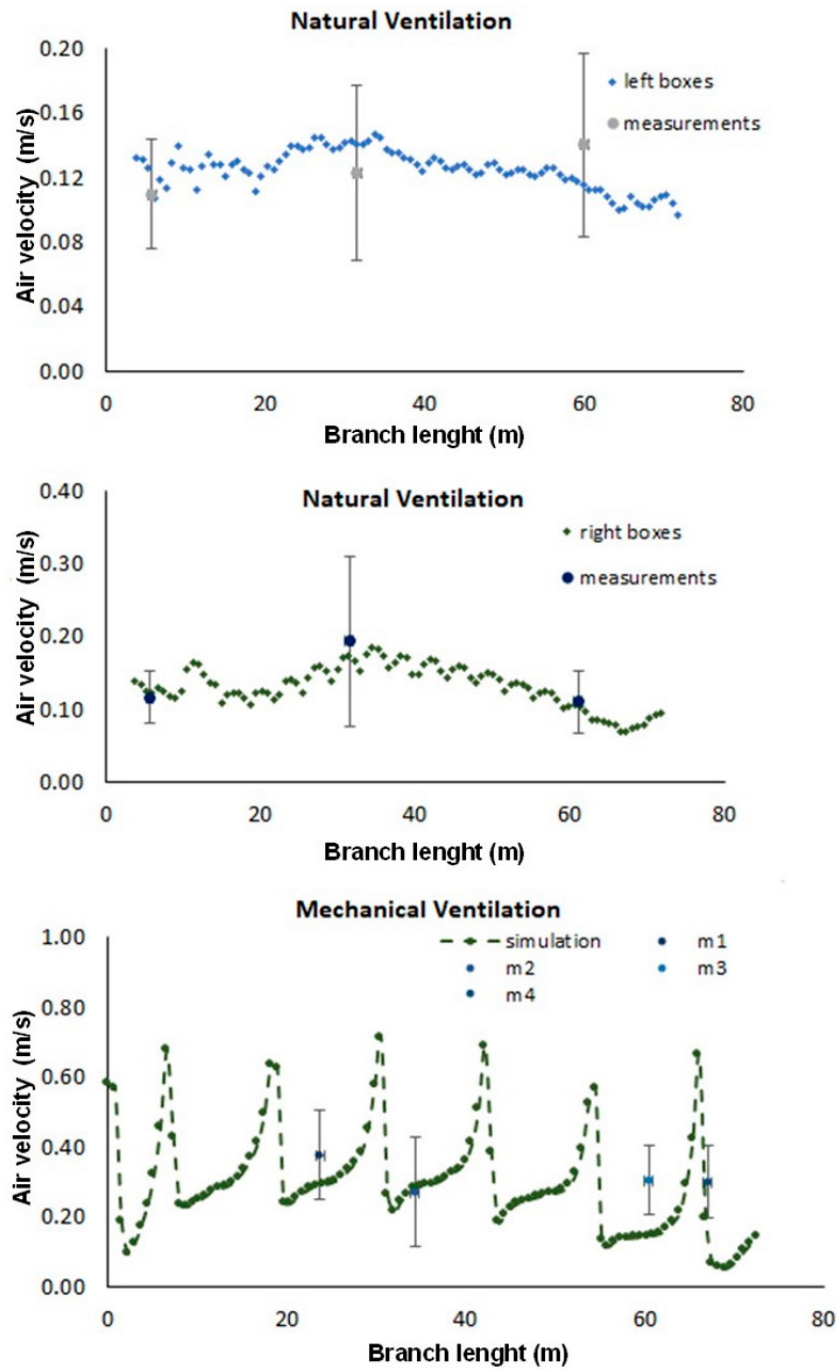

Supplement: Supplementary file 1 [file animals-11-02338-s001.zip › Supplementary figure S2.pdf]
